# Curved crystal morphology, photoreactivity and photosalient behaviour of mononuclear Zn(II) complexes

**DOI:** 10.1107/S2052252516019072

**Published:** 2017-01-01

**Authors:** Caroline Evania Mulijanto, Hong Sheng Quah, Geok Kheng Tan, Bruno Donnadieu, Jagadese J. Vittal

**Affiliations:** a Department of Chemistry, National University of Singapore, 3 Science Drive 3, Singapore 117543, Singapore

**Keywords:** cycloaddition reaction, curved single crystals, photosalient property, Zn(II) complex

## Abstract

A dramatic effect of crystal morphology, photoreactivity and photosalient property is observed in a zinc(II) complex due to solvent effects and fluorine substitution at the backbone of the ligand. Of the two crystal forms with a 3-fluoro derivative, one yielded a curved morphology of single crystals and the second form shows photoreactivity in the solid state, whereas crystals of the 2-fluoro derivative pop during the [2 + 2] photocycloaddition reaction. This is the first report documenting curved single crystals of metal complexes obtained naturally during crystallization, although such bent crystals have been observed in extended solids naturally, or bent by mechanical force or by UV irradiation.

## Abstract 2

Solid-state [2 + 2] photocycloaddition reactivity, mechanical motion during cycloaddition reaction under UV light and naturally curved single crystals are reported in structurally similar mononuclear Zn(II) coordination complexes containing fluoro derivatives of 4-styryl pyridine ligands.

## 1. Introduction   

A single crystal has a continuous crystal lattice of the entire sample with no grain boundaries and usually has straight edges, sharp corners and flat surfaces. On the contrary, the single crystals produced in biomineralization, such as bones, mollusk shells and teeth have unusual shapes with a size of about 100 µm (Addadi *et al.*, 1999 ▸). The arrested growth of nanocrystals by surfactants is known to produce unusually shaped crystals such as kinetic products (Camargo *et al.*, 2007 ▸; Han *et al.*, 2008 ▸; Li *et al.*, 2010 ▸; Tian *et al.*, 2010 ▸; Yu *et al.*, 2005 ▸). Further, several single crystals can be deformed by applying mechanical force (Ghosh & Reddy, 2012 ▸; Ghosh, Mishra, Ganguly *et al.*, 2015 ▸; Panda *et al.*, 2015 ▸; Reddy *et al.*, 2005 ▸; Uchida *et al.*, 2013 ▸; Varughese *et al.*, 2013 ▸). Photoinduced twisting, bending, curling, shape changing of single crystals of diarylethene (Irie, 2001 ▸; Morimoto & Irie, 2010 ▸; Kitagawa *et al.*, 2013 ▸; Kobatake *et al.*, 2007 ▸; Kuroki *et al.*, 2010 ▸; Terao *et al.*, 2012 ▸), anthracene derivatives (Al-Kaysi & Bardeen, 2007 ▸; Good *et al.*, 2009 ▸; Kim *et al.*, 2013 ▸, 2014 ▸; Zhu *et al.*, 2011 ▸, 2011*a* ▸, 2014 ▸), azobenzene derivatives (Bushuyev, Tomberg *et al.*, 2013 ▸; Bushuyev, Singleton & Barrett, 2013 ▸; Koshima & Ojima, 2012 ▸), olefin-based organic molecules (Sun *et al.*, 2013 ▸), salicylideneaniline (Koshima *et al.*, 2011 ▸) and other crystals and nanocrystals (Al-Kaysi *et al.*, 2006 ▸; Ghosh, Mishra, Kadambi *et al.*, 2015 ▸; Kim *et al.*, 2012 ▸; Zhang & Naumov, 2015 ▸) have been extensively investigated. Further, Naumov and co-workers (Panda *et al.*, 2014 ▸; Zhang *et al.*, 2016 ▸) studied thermoresponsive crystals. Single crystals of a number of non-molecular solids and inorganic compounds have been found to be helically twisted during crystallization forgoing their long-range order, with the crystals ranging in size from nanometers to centimeters. This subject has been reviewed in 2014 by Kahr and co-workers (Shtukenberg *et al.*, 2014 ▸). However, bent or curved single crystals formed naturally during a slow crystallization process without any external stimuli are rather rare. Recently a macrocyclic π-conjugated organic molecule was found to crystallize to produce curved single crystals due to phase contamination (Chou *et al.*, 2015 ▸). Indeed, single crystals of simple coordination complexes with curved crystal morphology naturally obtained during crystallization, to the best of our knowledge, have never been documented.

Here we report two polymorphs of a mononuclear Zn(II) complex of 3′-fluoro-4-styrylpyridine (3F-4spy), one with a lattice guest solvent, and the second form has no lattice solvent. The single crystals of the solvated crystals (polymorph I) have predominantly curved morphology, if grown from dimethylformamide (DMF) with tetrahydrofuran (thf) or acetonitrile solution. While the second polymorph, grown from DMF solution with normal rod-like crystal morphology, is photoreactive and the olefin groups of the 3F-4spy ligands undergo a solid-state [2 + 2] cycloaddition reaction under UV light. Packing of the Zn(II) complex of 2F-4spy is similar to that of the unsolvated polymorph, but the photoreactive single crystals exhibit photosalient properties, popping under UV light while undergoing a [2 + 2] cycloaddition reaction. The details of our investigations are described below.

## 2. Results and discussion   

### 2.1. Crystal structure of [Zn(NCS)_2_(4spy)_2_]·2MeOH (**1**)   

The complex [Zn(NCS)_2_(4spy)_2_]·2MeOH (**1**) crystallized in the monoclinic space group *Fdd*2 with *Z* = 8. The Zn(II) atom sits on the crystallographic twofold *c*-axis. The complex is packed along the shortest *c*-axis with a Zn⋯Zn distance of 5.1092 (5) Å which corresponds to the longest dimension of the crystals. Two NCS anions and two 4spy ligands are bonded to provide the Zn(II) atom a distorted tetrahedral ZnN_4_ core. The S atoms of the NCS ligands are closer to the neighbouring Zn(II) atoms with Zn⋯S distances of 5.06 Å, which is far greater than the sum of the van der Waals radii of 3.19 Å. As a result, the 4spy ligands are slip-stacked such that the Zn(II) atoms are closer to the centre of the neighbouring pyridyl rings with a distance of 4.03 Å. Further, the centre of the olefin bonds are in close proximity to the neighbouring pyridyl ring on the other side by 3.61 Å, as well as to the phenyl ring by 3.26 Å. The disordered methanol molecules occupy the channel created by the ligands along the *c*-axis.

### 2.2. Curved crystal morphology of [Zn(NCS)_2_(3F-4spy)_2_]·DMF (**2**)   

**Figure 1:**
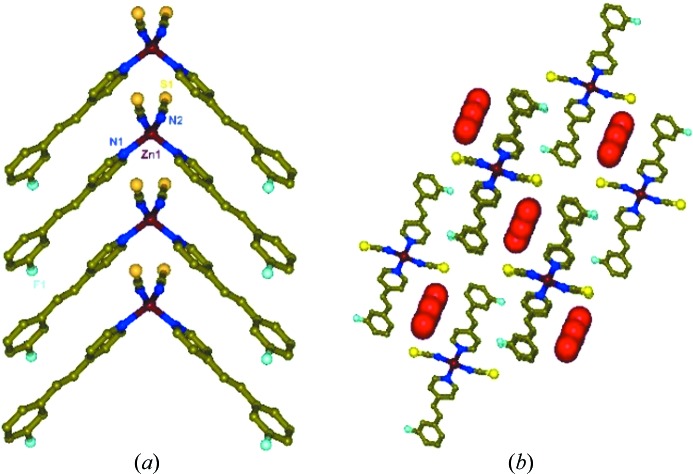
A view showing the packing of [Zn(NCS)_2_(3F-4spy)_2_] in (**2**) along the *b*-axis (*a*) and another view from the *b*-axis showing the channels occupied by guest solvents (*b*).

In the complex [Zn(NCS)_2_(3F-4spy)_2_]·DMF (**2**) which crystallized in the monoclinic space group *P*2/*n* with *Z* = 2, the asymmetric unit contains half the formula and Zn(II) is on the crystallographic *n*-glide plane. The packing is otherwise very similar to that of **1** (Fig. 1 ▸ *a*). All the one-dimensional aggregates are packed along the *b*-direction and generate channels subtended by arrays of NCS anions and 3F-4spy ligands, filled with highly disordered DMF guest molecules used for crystallization (Fig. 1 ▸ *b*).

**Figure 2:**
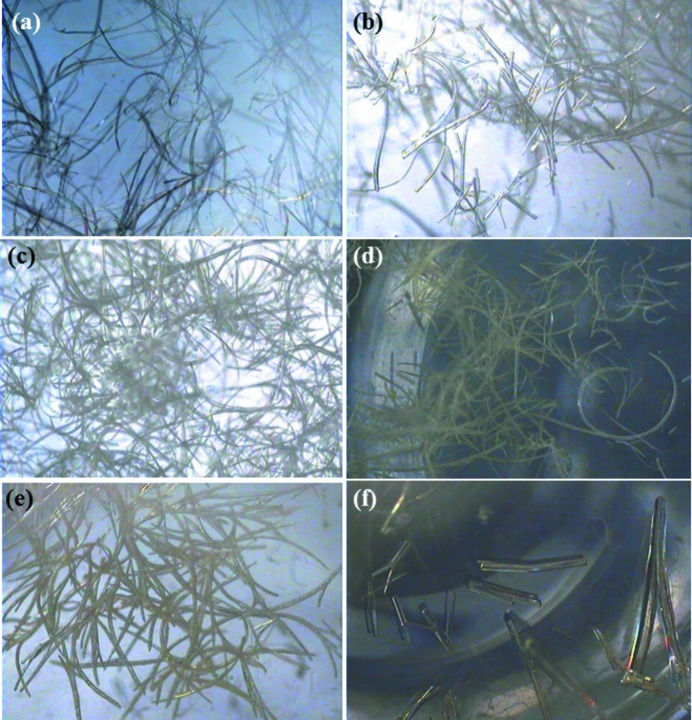
Curved single crystals of **2** in (*a*) DMF and thf; (*b*) DMF, acetonitrile and diethylether; (*c*) DMF, acetonitrile and thf; (*d*) DMF, acetonitrile and chloroform; (*e*) DMF, acetonitrile and ethylene glycol; (*f*) DMF and methanol.

Due to the short *b*-axis of 4.963 Å compared with *a* and *c* lengths, the crystals formed as long needles, predictably. The complexes are aligned as one-dimensional aggregates along the short *b*-axis. Crystallization from a dilute solution of DMF along with thf or acetonitrile or acetone solution yielded highly curved and bent single crystals, as shown in Fig. 2 ▸. They are not dendritic wire-like crystals sometimes observed during crystallization. In the concentrated solutions, only straight rods were formed in the majority with no tendency to form curved crystals. The curved crystals are stable in air for a week and slowly split into pieces as the guest solvents are lost. Although having very similar solid-state packing, no bending or curving was observed in the single crystals of **1** under the different crystallization conditions tried. Probably, the crystallization condition used for **1** did not provide very long and thin single crystals needed for bending.

Usually crystal defects like dislocations, epitaxial growth and grain boundaries will give rise to a branched growth of crystals with sharp bending (Benz & Neumann, 2014 ▸; Chernov, 1984 ▸). Well organized smooth curving at the macroscopic level probably requires distortions at the molecular level. Under dilute solution, thin and long rods are formed due to the arrested growth along the planes parallel to the *b*-axis. It is likely that the highly coordinating solvents such as DMF, acetonitrile or thf or acetone are able to bind to the surface of the {100} planes where the F atoms are exposed and probably weakly interact with the F atoms. In this process, these coordinating solvents, DMF and thf also bind to Zn(II) causing an increase in the coordination number and change in the coordination geometry which is probably responsible for bending and curving of the packing of the Zn(II) monomers. Hence, the short crystallographic *b*-axis with the longest dimension of the needle-shaped crystals starts bending and curving during crystallization. Inconsistency in the elemental analysis of the fresh single crystals appear to support this proposed model. The absence of very strong intermolecular interactions between the neighbouring one-dimensional aggregates as well as the presence of free void space close to these strands which are partially filled by disordered guest solvents appear to minimize the stress due to this curving of single crystals. Further, when the lattice solvents were removed by keeping the curved crystals at room temperature for several days, they split into pieces, probably due to the strain induced by the loss of guest solvents.

The defects at the molecular level have also been realised very recently in coordination polymers and metal–organic framework compounds (Al-Janabi *et al.*, 2016 ▸; Choi *et al.*, 2011 ▸; Fang *et al.*, 2015 ▸; Furukawa *et al.*, 2015 ▸; Sholl & Lively, 2015 ▸; Taylor *et al.*, 2015 ▸; Trickett *et al.*, 2015 ▸). Although identification and characterization of these defects arising from the substitution and vacancy of the spacer ligands are challenging, they are known to alter the gas sorption and catalytic properties. The usual single-crystal and powder X-ray diffraction techniques are very insensitive to these defects and are not useful to gain insights into the details. On a similar note, the curving of single crystals probably occurs due to molecular defects caused by the coordination environment at Zn(II) as discussed before, which influence the packing of one-dimensional aggregates of the Zn(II) complex monomers. More direct influences of the molecular defects on the other properties of the metal complexes may be waiting to be exploited.

### 2.3. Photoreactivity of the polymorph [Zn(NCS)_2_(3F-4spy)_2_]·DMF (**3**)   

**Figure 3:**
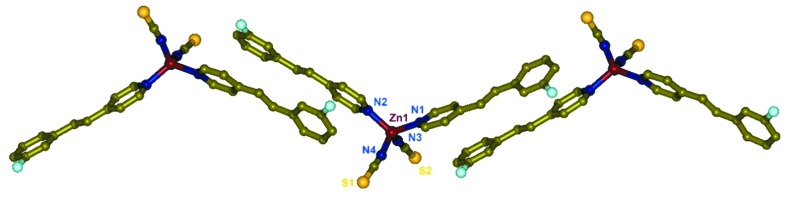
Packing of [Zn(NCS)_2_(3F-4spy)_2_] in **3** which promotes the formation of a one-dimensional coordination polymer upon [2 + 2] cycloaddition reaction under UV light.

The second polymorph was obtained without any guest solvent in the lattice. Single crystals of [Zn(NCS)_2_(3F-4spy)_2_] (**3**) obtained as blocky crystals from DMF and methanol solution crystallized in the monoclinic space group *P*2_1_/*c* with *Z* = 4, and the asymmetric unit has one formula unit. The neighbouring 3F-4spy ligands in the Zn(II) monomers are aligned in a head-to-tail manner as shown in Fig. 3 ▸ approximately along the 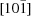 direction. The close non-bonding distances between the centres of the pyridyl rings to the 3F-phenyl groups are 3.77 and 3.73 Å, indicating the existence of π–π interactions. This enforces the olefin pairs to align closer with a distance of 3.79 Å but in an antiparallel manner (Schmidt, 1971 ▸). A quantitative [2 + 2] cycloaddition reaction takes place in the solid state under UV light in **3** as monitored by time-dependent ^1^H NMR spectroscopy. Based on the packing in Fig. 3 ▸, the final photoproduct can be predicted to be a one-dimensional coordination polymer (one-dimensional CP) with the spacer ligand *rctt*-1,3-bis­(4-pyridyl)-2,4-bis­(3′-fluorophenyl)cyclobutane) (*rctt*-3F-ppcb) after the pedal motion of the olefin groups (Medishetty *et al.*, 2013 ▸; Medishetty, Bai *et al.*, 2015 ▸). Unfortunately, this is not a single-crystal-to-single-crystal reaction. The pseudopolymorphs **2** and **3** exhibit two different solid-state properties, namely, curved single crystals and photoreactivity, respectively.

### 2.4. Photosalient property of [Zn(NCS)_2_(2F-4spy)_2_] (**4**)   

Photoinduced mechanical motions of single crystals including crawling, rotating, curling, leaping, jumping, hopping, popping, splitting and exploding are interesting phenomena and recently attracted attention due to their potential conversion of light energy to mechanical energy (Hayashi & Koizumi, 2016 ▸; Hoshima *et al.*, 2011 ▸; Koshima *et al.*, 2009 ▸; Kim *et al.*, 2012 ▸, 2013 ▸; Kitagawa *et al.*, 2013 ▸; Kobatake *et al.*, 2007 ▸; Morimoto & Irie, 2010 ▸; Naumov *et al.*, 2010 ▸, 2013 ▸, 2015 ▸; Terao *et al.*, 2012 ▸; Uchida *et al.*, 2008 ▸; Zhu *et al.*, 2011 ▸). Such a property has also been observed in metal complexes while undergoing [2 + 2] cycloaddition reactions under UV light (Medishetty *et al.*, 2014 ▸; Medishetty, Bai *et al.*, 2015 ▸). The metal complex of 4spy ligands and their fluoro derivatives have been found to be useful for this purpose. Here we found that the crystals of [Zn(NCS)_2_(2F-4spy)_2_] (**4**) pop under UV light during a [2 + 2] cycloaddition reaction. The details are described below.

**Figure 4:**
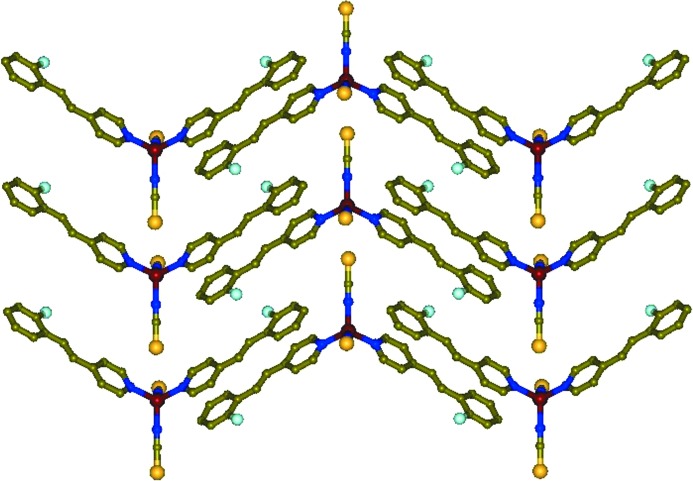
One-dimensional packing of photosalient [Zn(NCS)_2_(2F-4spy)_2_], **4**.

In **4**, which crystallized in the orthorhombic space group *Pnma* with *Z* = 4, the monomer has a crystallographic mirror plane containing the Zn(NCS)_2_ fragment. Two 2F-4spy ligands are bonded to provide an ideal tetrahedral geometry at Zn(II). In the 2F-4spy ligand, the two rings are twisted away by 10.8° from planarity. The neighbouring 2F-4spy ligands are aligned in a head-to-tail fashion as shown in Fig. 4 ▸. The distance of 3.80 Å between the two rings in the 2F-4spy ligand pairs indicates the presence of π–π interactions. Further, the centre-to-centre distance between the olefin pairs is 3.83 Å. Based on Schmidt’s topochemical criteria, **4** is expected to be photoreactive (Schmidt, 1971 ▸). Indeed, UV irradiation of powdered **4** undergoes quantitative photoreaction leading to the formation of *rctt*-2F-ppcb as monitored by the ^1^H NMR spectral data from the appearance of a cyclobutane peak at 4.60 p.p.m. and new pyridyl peak at 8.30 p.p.m. for the *rctt*-2F-ppcb ligand. Overall its photoreactivity is very similar to that of **3**.

Interestingly, the single crystals of **4** pop violently when they were exposed to UV light. The photomechanical behaviour is very similar to those reported before and the single crystals were broken into pieces while flying apart (Medishetty *et al.*, 2014 ▸; Medishetty, Sahoo *et al.*, 2015 ▸). The percentage of change in density, 17.6%, during this dimerization process is on the higher side of the range observed before. Further, the first-order rate constant (0.1786 min^−1^) for the dimerization process is higher than the other Zn(II) complexes (Medishetty *et al.*, 2014 ▸). Close examination of the crystal packing shown in Fig. 4 ▸ reveals that the olefin pairs run approximately parallel to the [110] and [1ī0] axes and hence the formation of cyclobutane rings and the associated conformational changes are expected to create strain along the direction of the *a*-axis, the longest dimension of the crystal. A rapid phase transition of **4** into photoproducts facilitates the conversion of the accumulated strain energy into kinetic energy. A comparison of the packing between **3** and **4** revealed that a sudden anisotropic volume increase cannot occur in **3** and hence **3** is not popping under UV light. This is also supported by the fact that the change in the percentage of change in density during the formation of one-dimensional CP from **3** is only 11.4%.

## 3. Conclusions   

In summary, fluorine substitution at different positions of the phenyl ring of the ligand in the mononuclear Zn(II) complex has a great effect on their solid-state properties. The results are summarized in Scheme 1. Such influence of fluoro substitution on the isomerization as well as photoreactivity has been noted before (see, for example, Bléger *et al.*, 2012 ▸; Bushuyev, Tomberg *et al.*, 2013 ▸). The formation of curved single crystals of **2** could be attributed to this effect. Although both **3** and **4** exhibit photoreactivity, only **4** has the photosalient property due to packing which promotes sudden anisotropic volume expansion to relieve the stress during the photoreaction. It is quite surprising that a small variation in the crystallization conditions and substitution at the backbone of the 4spy ligand made huge changes in the morphology of single crystals, interesting influence on photoreactivity and dramatic impact on photosalient properties in a series of structurally closely related monomeric Zn(II) complexes. Understanding these changes at the molecular level on the new physical properties and chemical reactivity will certainly lead to the development of advanced functional and smart materials with exciting new properties. More systematic study and analysis of how fluoro substitution affects the photosalient behaviour are in progress.
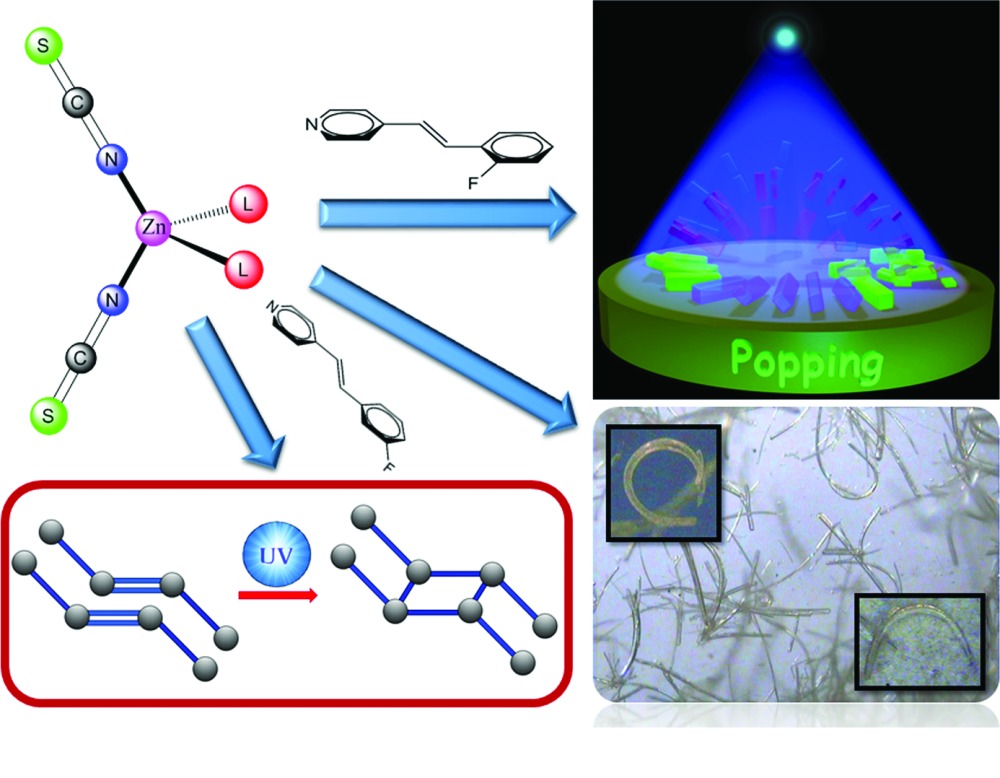


## 4. Experimental   

### 4.1. Materials and general methods   

All the chemicals and solvents were of reagent or better grade purchased from different commercial resources and used without further purification unless mentioned. Powder X-ray diffraction (PXRD) data were recorded on a D5005 Siemens X-ray diffractometer with graphite monochromated Cu *K*α radiation (λ = 1.54056 Å) at room temperature (298 K). NMR spectra were recorded on a 300 MHz Bruker Avance 300 FT-NMR spectrometer by calibrating the residual solvent as the reference in DMSO-*d*_6_ solution. Thermogravimetric analysis (TGA) was performed under a nitrogen atmosphere with a heating rate of 5°C min^−1^ on a TA instruments SDT-2960. The C, H, N analysis was carried using an Elementar Vario Micro Cube instrument at the Elemental Analysis Lab, CMMAC, Department of Chemistry, National University of Singapore. The UV irradiation experiments were conducted in a LUZCHEM UV reactor. In the case of the percentage of photo-products formed *versus* time plots, the ground single crystals were packed between the Pyrex glasses and placed in the UV reactor. These glass slides were flipped back at regular intervals of time to maintain uniform exposure of UV light. The samples were taken out at regular intervals of time and dissolved in DMSO-*d*_6_ to record ^1^H NMR spectra to follow the course of the reaction.

### 4.2. Synthesis of [Zn(NCS)_2_(4spy)_2_]·2MeOH (**1**)   

Colourless block-like single crystals were obtained from slow evaporation of 2 ml of a methanolic solution of Zn(NO_3_)_2_·6H_2_0 (7.5 mg, 0.025 mmol), KSCN (5 mg, 0.05 mmol) and 4spy (9 mg, 0.05 mmol) and dried at room temperature. Yield: 62%. ^1^H NMR (DMSO-*d*_6_, 300 MHz, 298 K): δ = 8.54 (d, 4H, pyridyl proton of 4spy), 7.23–7.68 (m, 18H, aromatic protons of 4spy). Elemental analysis: calculated for the desolvated **1**: C_28_H_22_N_4_S_2_Zn (544.01): C 61.82, H 4.08, N 10.30; found: C 61.57, H 4.23, N 10.21%.

All the solvent combinations and conditions used to obtain the curved crystals of **2** have been tried for **1**. However, all these conditions only yielded long rods. No curved crystals were observed in the bulk. The details are given in the supporting information (Fig. S1).

### 4.3. Synthesis of [Zn(NCS)_2_(3F-4spy)_2_]·DMF (**2**)   

Bent crystals were obtained from slow evaporation of acetonitrile (2 ml) or thf (2 ml) or acetone (2 ml) and a DMF (1 ml) solution of Zn(NO_3_)·6H_2_O (7.5 mg, 0.025 mmol), KSCN (5.0 mg, 0.05 mmol) and 3F-4spy (10 mg, 0.05 mmol) and dried at room temperature. Yield: 68%. ^1^H NMR (DMSO-*d*_6_, 300 MHz, 298 K): δ 8.55 (d, 4H, pyridyl proton of 4spy), 7.12–7.57 (m, 16H, aromatic protons of 3F-4spy). Elemental analysis for the desolvated **2**: C_28_H_20_F_2_N_4_S_2_Zn (579.97): C 57.98, H 3.48, N 9.66; found: C 57.31, H 4.12, N 9.60%. More details and photographs are shown in the supporting information (Fig. S2).

### 4.4. Synthesis of straight [Zn(NCS)_2_(3F-4spy)_2_] (**3**)   

Colorless block single crystals were obtained from slow evaporation of MeOH (2 ml) or ethanol (2 ml) and DMF (1 ml) solution of Zn(NO_3_)·6H_2_O (7.5 mg, 0.025 mmol), KSCN (5.0 mg, 0.05 mmol) and 3F-4spy (10 mg, 0.05 mmol) and dried at room temperature. Alternatively, it can be synthesized with (2 ml) DMF only. Yield: 51%. ^1^H NMR (DMSO-*d*_6_, 300 MHz, 298 K): δ 8.55 (d, 4H, pyridyl proton of 4spy), 7.12–7.57 (m, 16H, aromatic protons of 3F-4spy). Elemental analysis for C_28_H_20_F_2_N_4_S_2_Zn (579.97): C 57.98, H 3.48, N 9.66; found: C 57.98, H 3.40, N 9.68%.

Our attempts to obtain single crystals of the photoproduct from **3** were unsuccessful.

### 4.5. Synthesis of [Zn(NCS)_2_(2F-4spy)_2_] (**4**)   

Yellow block single crystals were obtained from slow evaporation of DMF (1 ml) solution of Zn(NO_3_)_2_·6H_2_0 (7.5 mg, 0.025 mmol), KSCN (5 mg, 0.05 mmol) and 2F-4spy (10 mg, 0.05 mmol) and dried at room temperature. Yield: 57%. ^1^H NMR (DMSO-*d*_6_, 300 MHz, 298 K): δ 8.59 (d, 4H, pyridyl proton of 4spy), 7.00–7.86 (m, 16H, aromatic protons of 4spy). Elemental analysis: calc. for C_28_H_20_F_2_N_4_S_2_Zn (579.97): C 57.98, H 3.48, N 9.66; found: C 57.84, H 3.56, N 9.62%.

Our attempts to obtain single crystals of the photoproduct from **4** were unsuccessful.

### 4.6. X-ray crystallography   

Crystal data of all these crystals were collected on a Bruker AXS D8 Venture equipped with a Photon 100 CMOS active pixel sensor detector using graphite-monochromated Mo *K*α radiation (λ = 0.71073 Å) using a sealed tube. Absorption corrections were made with the program *SADABS* (Sheldrick, 1996 ▸), and the crystallographic package *SHELXTL* (Sheldrick, 2008 ▸; Müller *et al.*, 2006 ▸) was used for all calculations. CCDC 1474516–1474519 contains the supplementary crystallographic data for this paper, which can be obtained free of charge from the Cambridge Crystallographic Data Centre *via* www.ccdc.cam.ac.uk/data_request/cif.

#### 4.6.1. Crystal data for **1** at 100 (2) K   

C_30_H_30_N_4_O_2_S_2_Zn, *FW* = 608.07, orthorhombic, *Fdd*2; *a* = 36.663 (3), *b* = 37.055 (4), *c* = 5.1092 (5) Å, *V* = 6940.8 (11) Å^3^, *Z* = 8, ρ_calc_ = 1.164 g cm^−3^, μ = 0.857 mm^−1^, *GOF* = 1.186, final *R*_1_ = 0.0887, *wR*_2_ = 0.2644 [for 3071 data *I* > 2σ(*I*)]. Flack parameter = 0.024 (13). One methanol guest solvent in the asymmetric unit was found to be disordered. Two disorder models were refined and the occupancy was refined to 0.47 (3).

#### 4.6.2. Crystal data for **2** at 100 (2) K   

C_31_H_27_F_2_N_5_OS_2_Zn, *FW* = 653.10, monoclinic, *P*2/*n*; *a* = 14.871 (2), *b* = 4.9627 (9), *c* = 20.315 (3) Å, β = 93.183 (4)°, *V* = 1496.9 (4) Å^3^, *Z* = 2, *r*_calc_ = 1.287 g cm^−3^, μ = 0.994 mm^−1^, *GOF* = 0.998, final *R*_1_ = 0.0610, *wR*_2_ = 0.1114 [for 2098 data *I* > 2σ(*I*)]. *PLATON* indicates the Total Potential Solvent Accessible Void Volume, 235.9 Å^3^ (15.8%) for the total cell volume 1496.9 Å^3^. First we tried to model the highly disordered solvent region. The scattered electron densities were assigned to 9 C atoms with occupancies of 0.5, of which one was sitting at the origin. The individual isotropic displacement parameters were refined. The model refined to *R*1 = 0.0648 for 2059 reflections *F*_o_ > 4σ(*F*_o_) and 0.1455 for all 3550 data and 201 parameters with 0 restraints. But from the connectivity we were not able to recognize any guest DMF molecule. The electron densities in this disordered and unrecognizable solvent region were squeezed out of the *hkl* data using *PLATON* for further refinements. The model was refined satisfactorily with the squeezed data.

#### 4.6.3. Crystal data for **3** at 100 (2) K   

C_28_H_20_F_2_N_4_S_2_Zn, *FW* = 579.97, monoclinic, *P*2_1_/*c*; *a* = 10.3095 (4), *b* = 12.2083 (4) Å, *c* = 20.7566 (7) Å, β = 96.680 (1)°, *V* = 2594.72 (16) Å^3^, *Z* = 4, *r*_calc_ = 1.485 g cm^−3^, μ = 1.147 mm^−1^, *GOF* = 1.027, final *R*_1_ = 0.0361, *wR*_2_ = 0.0851 [for 5403 data *I* > 2σ(*I*)]. It appears that this ligand N1—C13 could be disordered. We tried to find a disorder model of this ligand and refine it with less than 8% occupancy. Since the overall quality is not improved, we kept the ‘no disorder’ structure.

#### 4.6.4. Crystal data for **4** at 100 (2) K   

C_28_H_20_F_2_N_4_S_2_Zn, *FW* = 579.97, orthorhombic, *Pnma*; *a* = 8.276 (5), *b* = 24.824 (15), *c* = 12.523 (8) Å, *V* = 2573 (3) Å^3^, *Z* = 4, ρ_calc_ = 1.497 g cm^−3^, μ = 1.157 mm^−1^, *GOF* = 1.079, final *R*_1_ = 0.0431, *wR*_2_ = 0.1070 [for 2712 data *I* > 2σ(*I*)]. The *ortho* F atom is disordered. The occupancy was refined to 0.913 (3).

## Supplementary Material

Crystal structure: contains datablock(s) d739, f076, f245, e286. DOI: 10.1107/S2052252516019072/lq5002sup1.cif

Structure factors: contains datablock(s) d739. DOI: 10.1107/S2052252516019072/lq5002d739sup2.hkl

Structure factors: contains datablock(s) f076. DOI: 10.1107/S2052252516019072/lq5002f076sup3.hkl

Structure factors: contains datablock(s) f245. DOI: 10.1107/S2052252516019072/lq5002f245sup4.hkl

Structure factors: contains datablock(s) e286. DOI: 10.1107/S2052252516019072/lq5002e286sup5.hkl

Supporting figures and tables. DOI: 10.1107/S2052252516019072/lq5002sup6.pdf

CCDC references: 1474516, 1474517, 1474518, 1474519
