# Supplementary material for: Curved crystal morphology, photoreactivity and photosalient behaviour of mononuclear Zn(II) complexes
Source: IUCrJ. 2017 Jan 1;4(Pt 1):65–71. doi: 10.1107/S2052252516019072 (PMC5331466; doi:10.1107/S2052252516019072)
Supplement: Supplementary file 6 [file m-04-00065-sup6.pdf]

IUCrJ

Volume 4 (2017)

Supporting information for article:

Curved crystal morphology, photoreactivity and photosalient behaviour of mononuclear Zn(II) complexes

Caroline Evania Mulijanto, Hong Sheng Quah, Geok Kheng Tan, Bruno Donnadieu and Jagadese J. Vittal

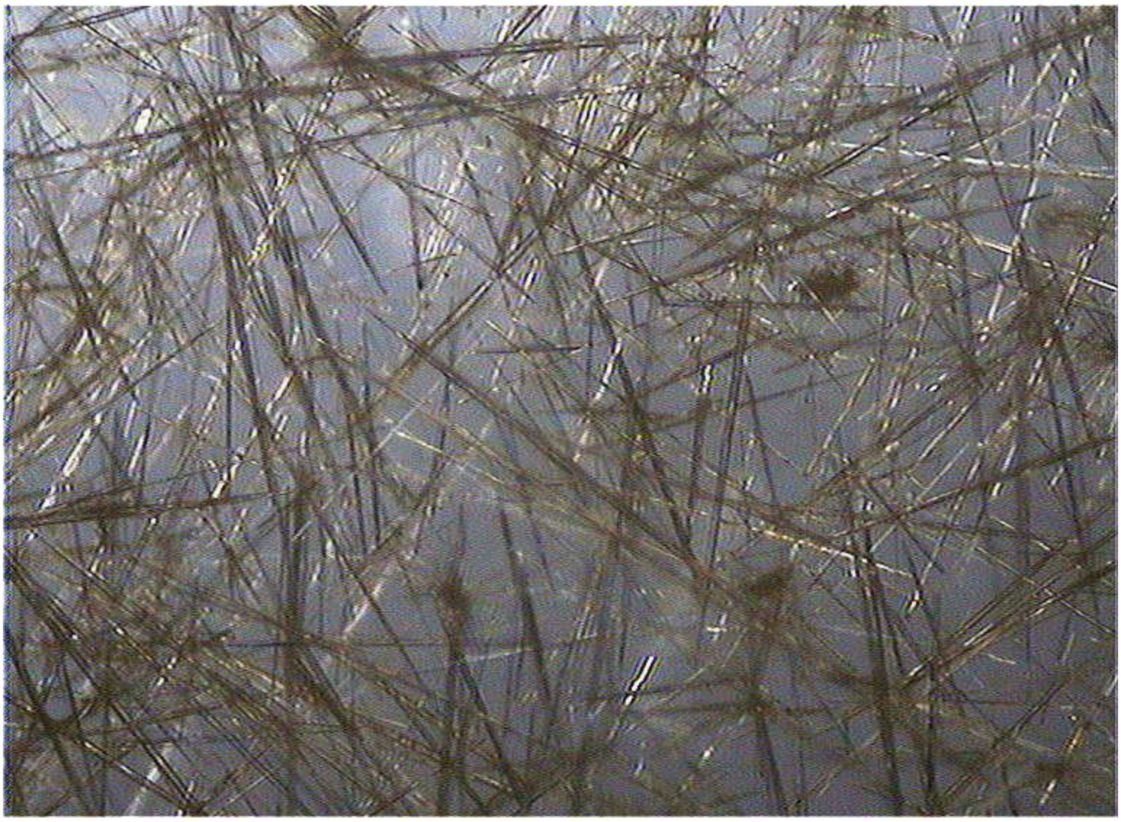

Figure S1 A typical photographs of crystals of **1** taken under microscope. All the solvent combinations and conditions used to get the curved crystals of **2** has been tried for **1**. But all these conditions yielded only long rods as shown in Figure S1. No curved crystals were observed in the bulk.

Solvent	Crystals	Type	Solvent	Crystals	Type
DMF/acetone	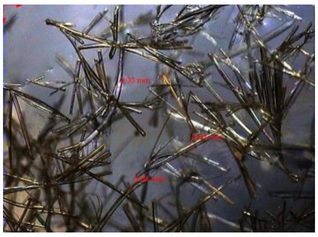	Bent Crystals	DMF only	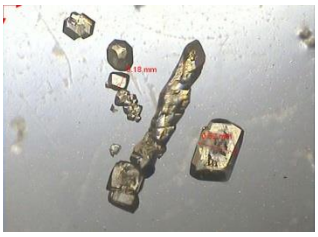	Blocky crystals
DMF/THF	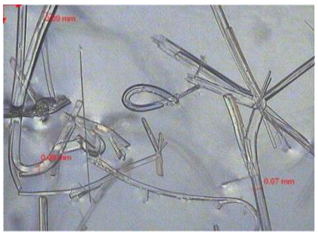	Bent crystals	DMF/MeOH	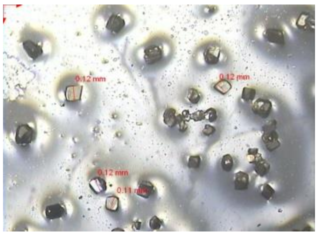	Straight crystals
DMF/ Acetonitrile	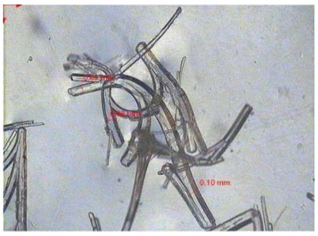	Bent crystals	DMF/EtOH	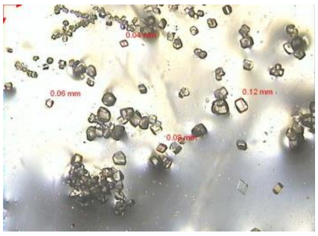	Straight crystals

Figure S2 Crystals of [Zn(NCS)2(3F-4spy)2]·under different solvent crystallization conditions. More photographs of curved crystals are appended at the end.

Table S1 Density measurements of the photoproducts

Density of the compounds were measured by floatation method using hexane (density: 0.6548 g cm^{-3}) and tetrachloroethylene (TCE, density: 1.62 g cm^{-3}).

**Compound**	**Density (experimental), g cm^{-3}**	**Density calculated from X-ray data, g cm^{-3}**	**Change in density (%)**
**3**	1.49(2)	1.485	11.4
**UV product 3a**	1.32(2)	NA	
**4**	1.49(2)	1.497	17.6
**UV product 4a**	1.22(2)	NA	

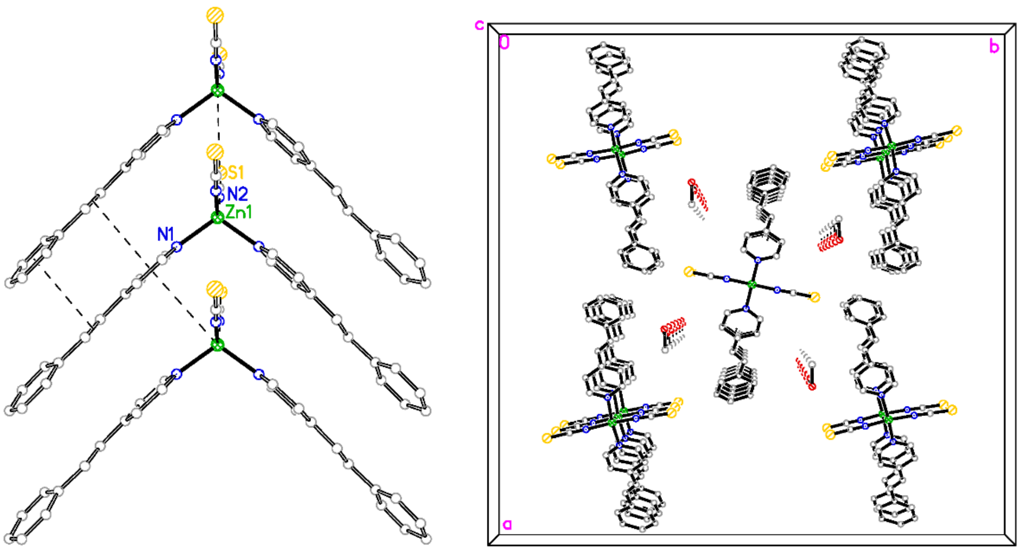

(a) **(b)**

Figure S3 (a) A view of the aggregation of the Zn(II) complex **1** along the *c*-axis (longest dimension of the crystal). (b). A view of the packing of the solvents in voids viewed from *c*-axis. PLATON indicates the Total Potential Solvent Accessible Void Volume, 2212.0 Å^3 (31.9%) for the total cell volume 6940.8 Å^3 which is quite unusual.

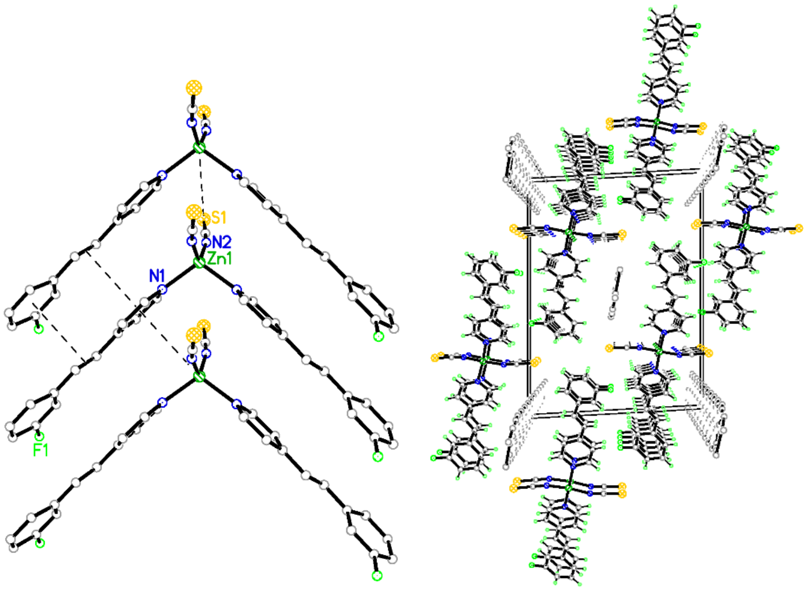

(a) (b)

Figure S4 (a) The packing of **2** is very similar to **1** although the space groups are different. The Zn(II) complex aggregates along *b*-axis. (b) Packing of Zn(II) along with solvent. PLATON indicates the Total Potential Solvent Accessible Void Volume, 235.9 Å^3 (15.8%) for the total cell volume 1496.9 Å^3.

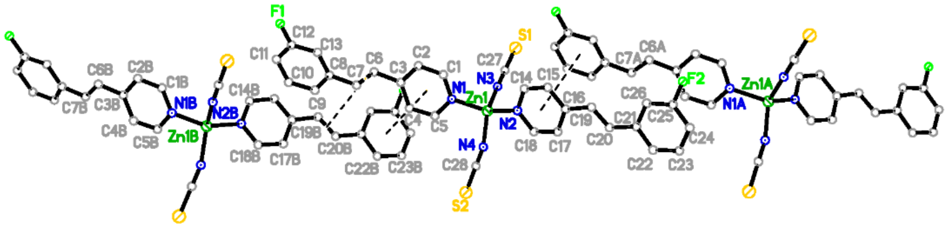

(a)

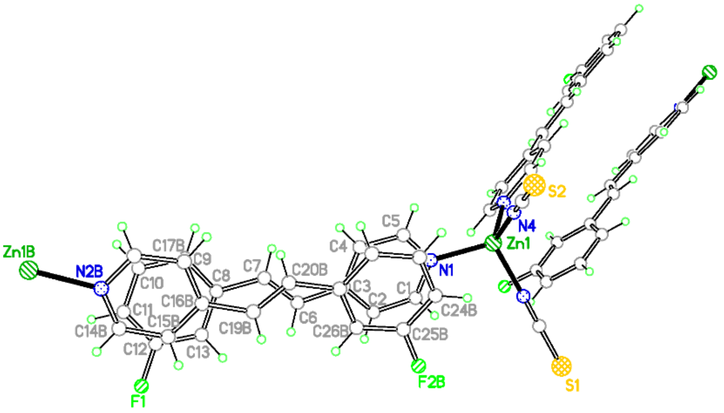

(b)

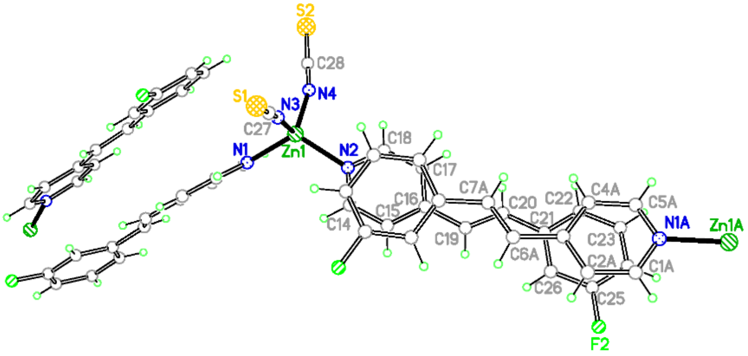

(c)

Figure S5 (a) The Alignment of 3F-4spy ligands in **3** are highlighted. (b) & (c) The crisscrossed alignments of the olefin pairs on both sides in **3**. The olefin pairs on both sides undergo pedal motion under UV light before reacting as inferred by ^{1}H-NMR data.

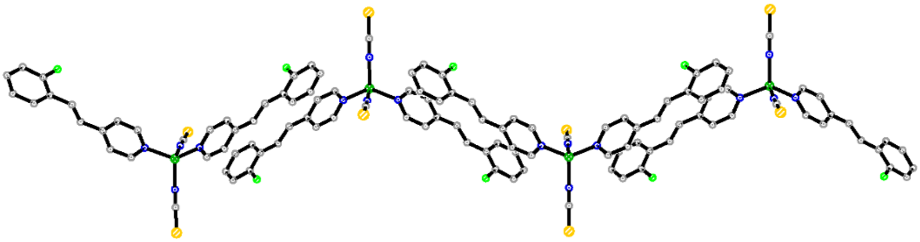

Figure S6 Packing of **4** showing the head-to-tail alignment of the 2F4spy ligands.

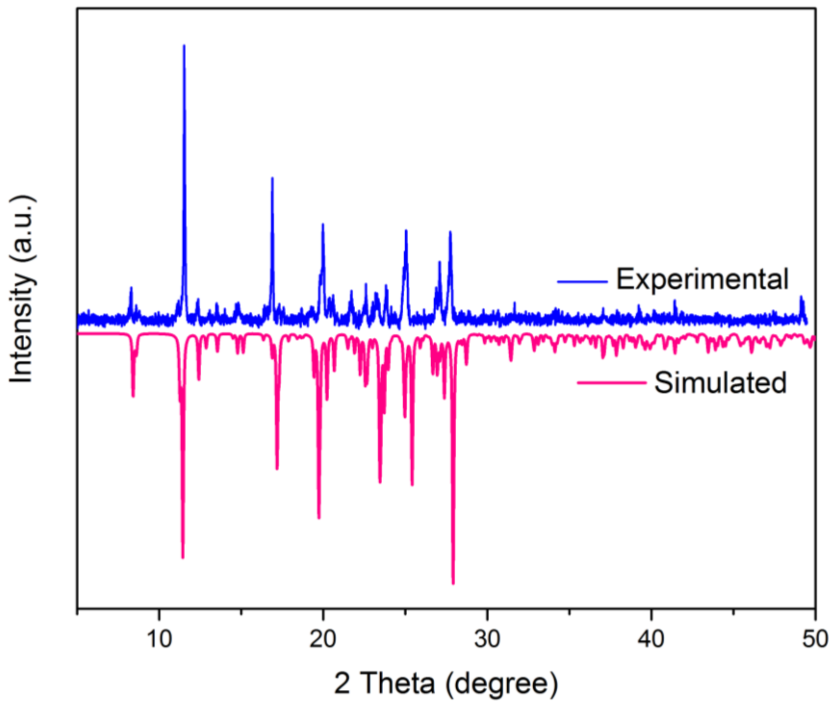

Figure S7 Comparison of PXRD patterns of compound **1.**

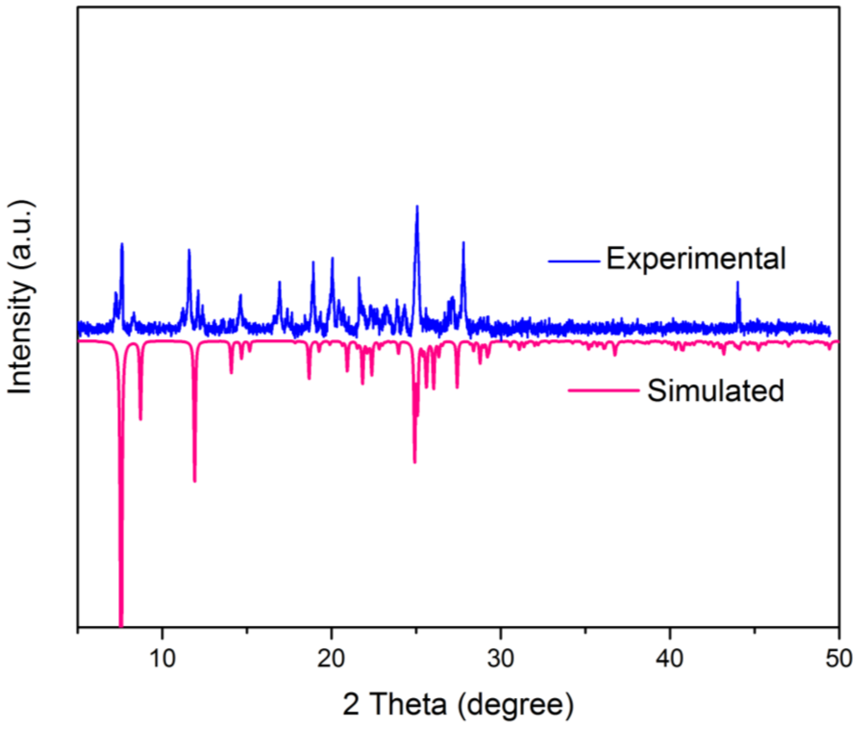

Figure S8 Comparison of PXRD patterns of compound **2**. Small difference could be due to the loss of solvents and hence the change in crystalline phase during grinding. A mixture of both normal and curved crystals were used.

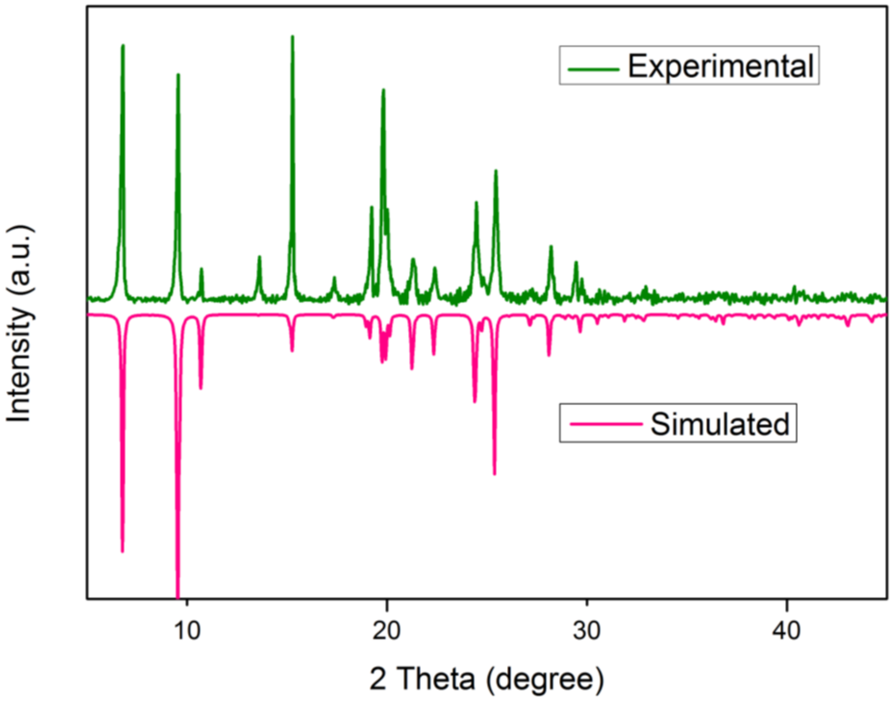

Figure S9 Comparison of PXRD patterns of compound **3**.

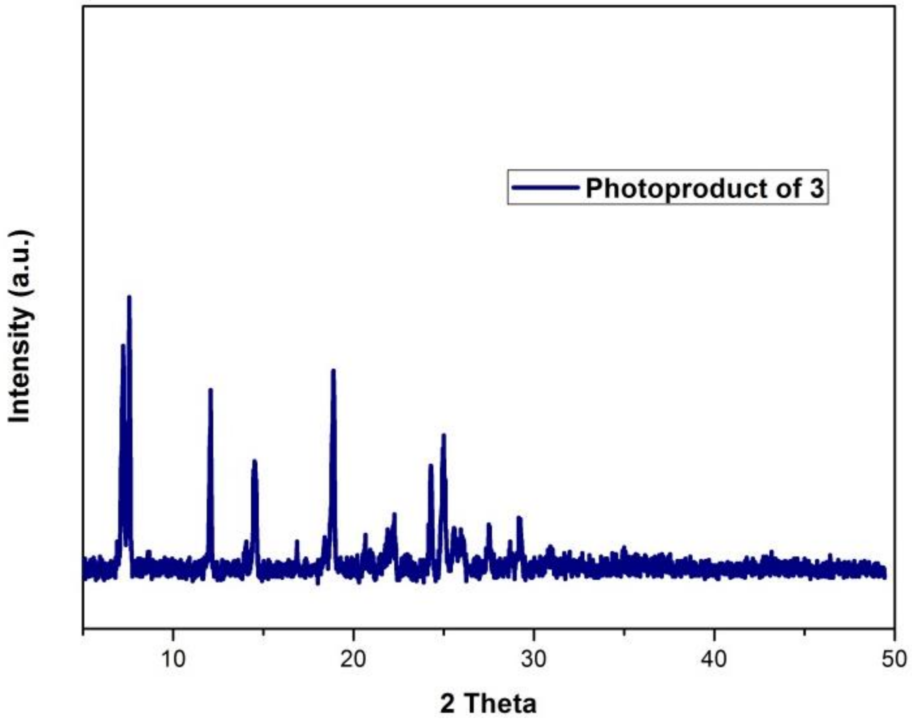

Figure S10 The PXPRD pattern of the photoproduct of **3** shows high crystallinity after the photoreaction.

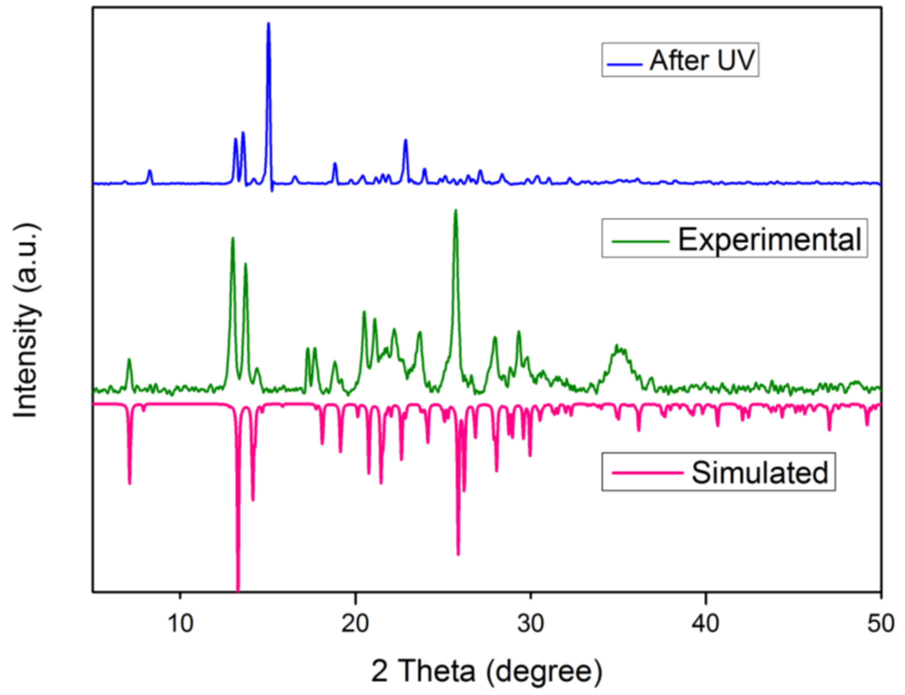

Figure S11 Comparison of PXRD patterns of compound **4** and its photoproduct after UV irradiation.

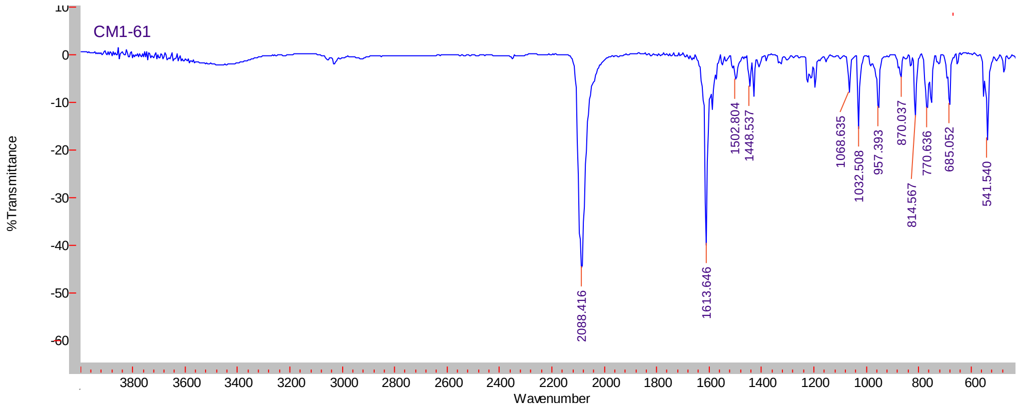

Figure S12 IR of [Zn(NCS)$_2$(4spy)$_2$]·2MeOH], **1.**

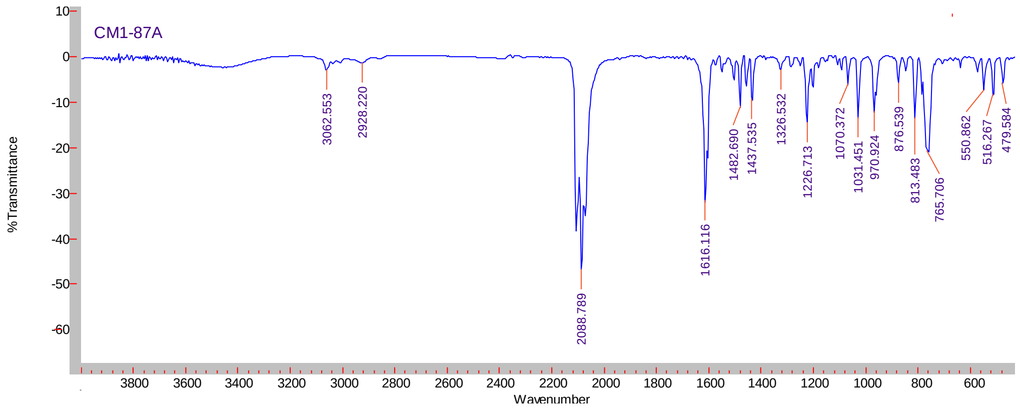

Figure S13 IR of $[Zn(NCS)_2(2F\text{-}4spy)_2]$, **4** before UV.

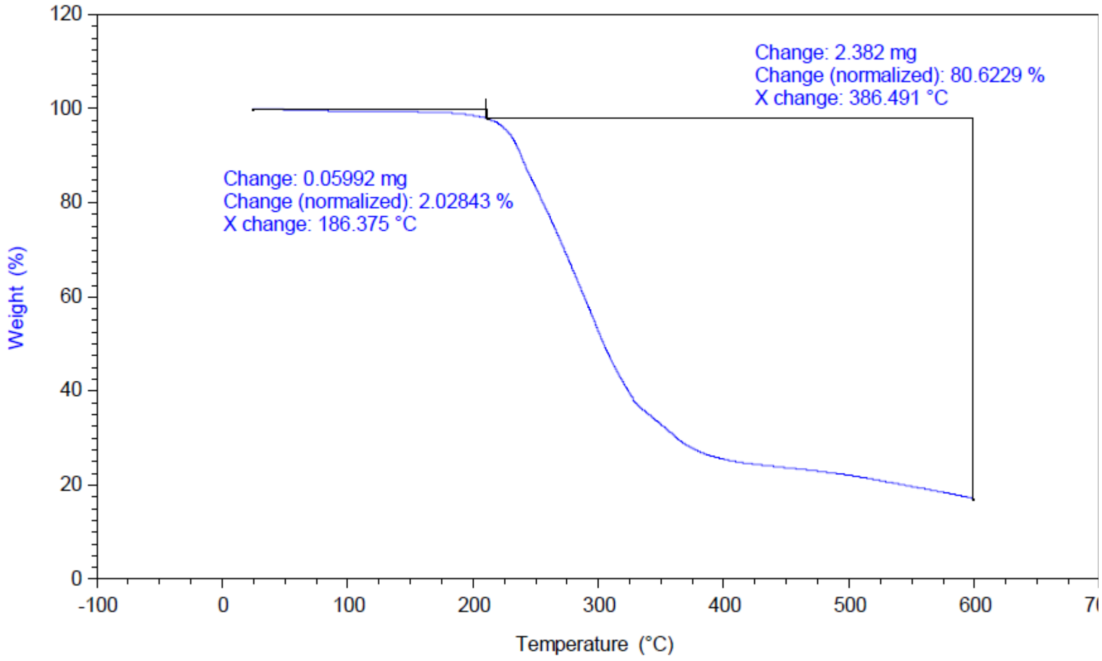

Figure S14 TGA of dried $[Zn(NCS)_2(4spy)_2]$, **1.**

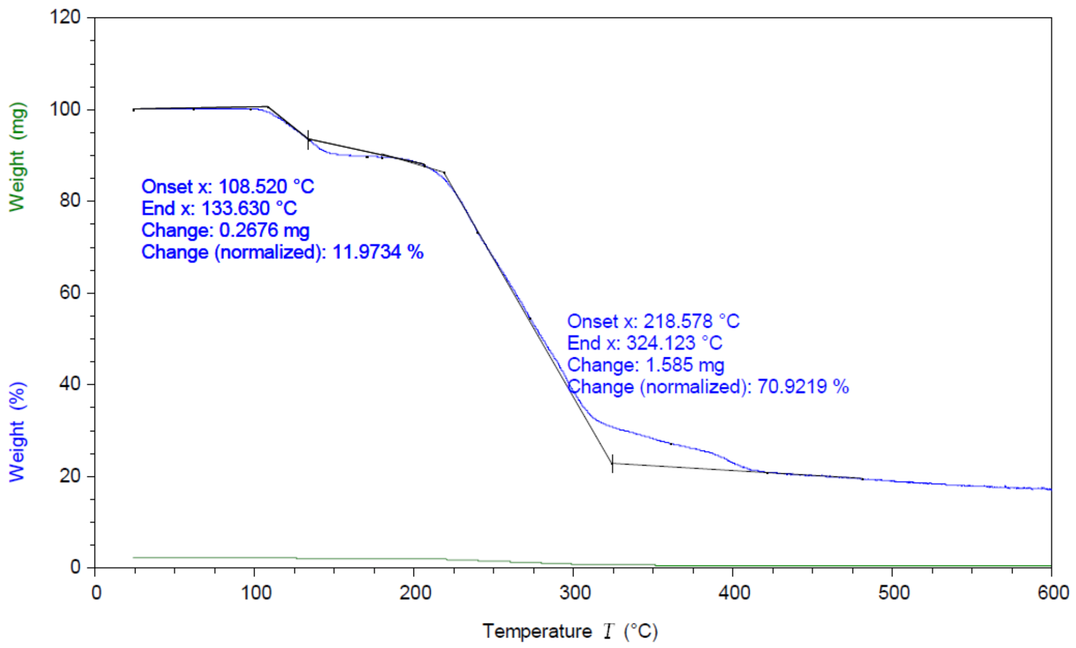

Figure S15 TGA of [Zn(NCS)$_2$(3F-4spy)$_2$]·DMF, **2**. MW = 653.10. This weight loss of 11.9% corresponds to the loss of one DMF molecule.

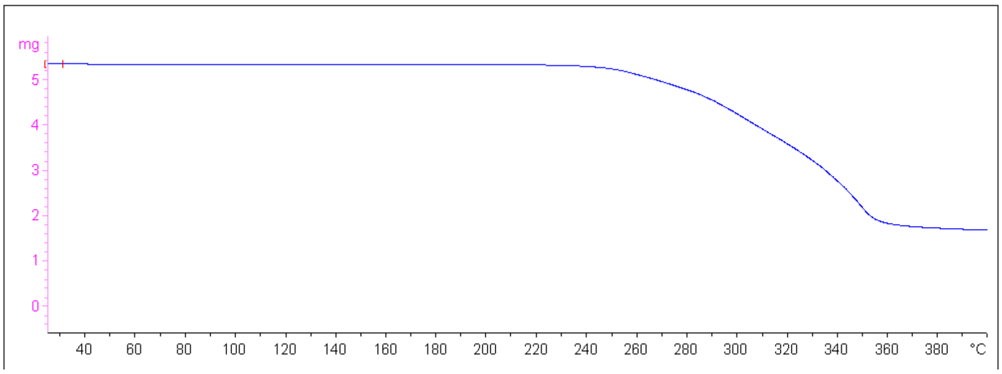

Figure S16 TGA of [Zn(NCS)$_2$(3F-4spy)$_2$], **3.**

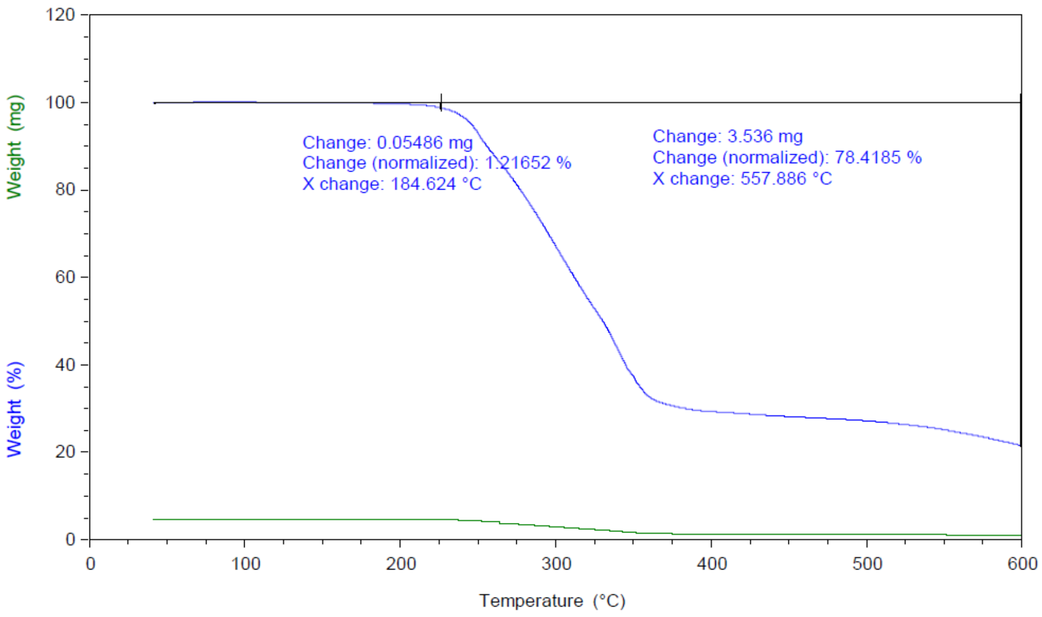

Figure S17 TGA of [Zn(NCS)$_2$(2F-4spy)$_2$], **4.**

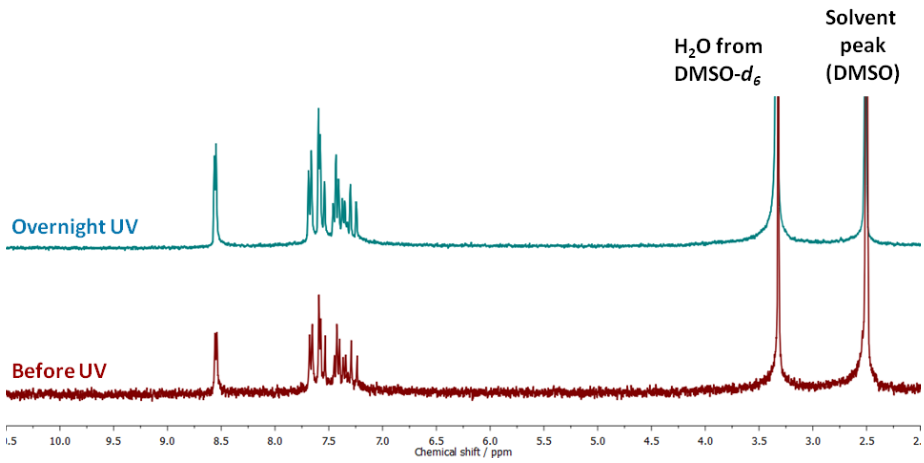

Figure S18 ^{1}H-NMR spectra of **2** before and after UV showing that this not photoreactive.

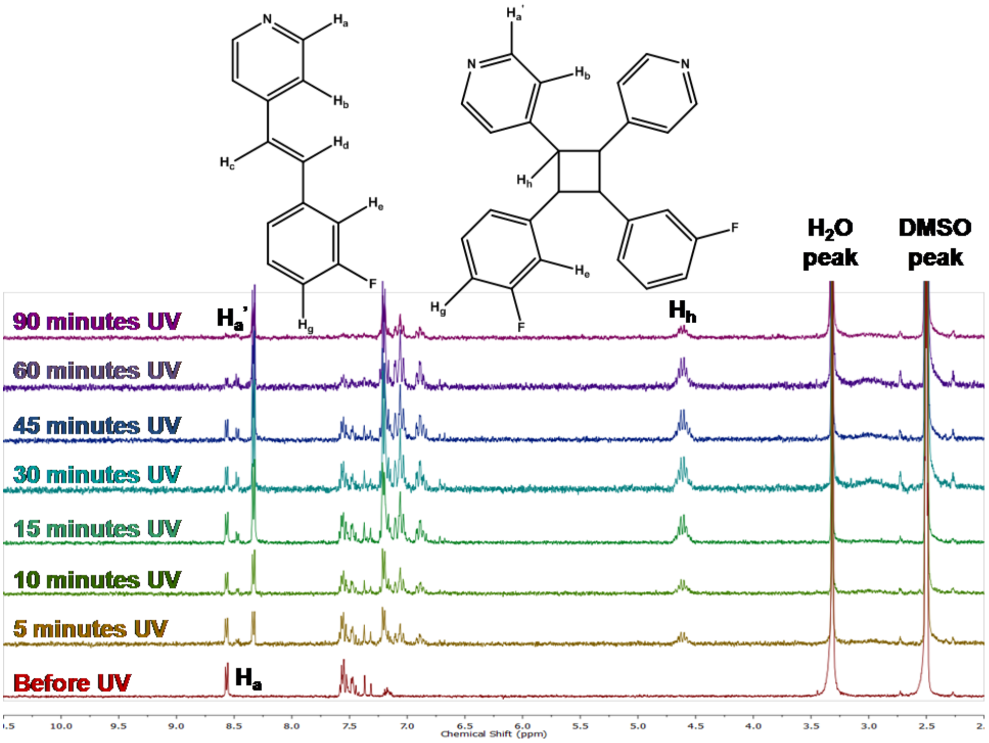

Figure S19 Time dimerization plot of **3**.

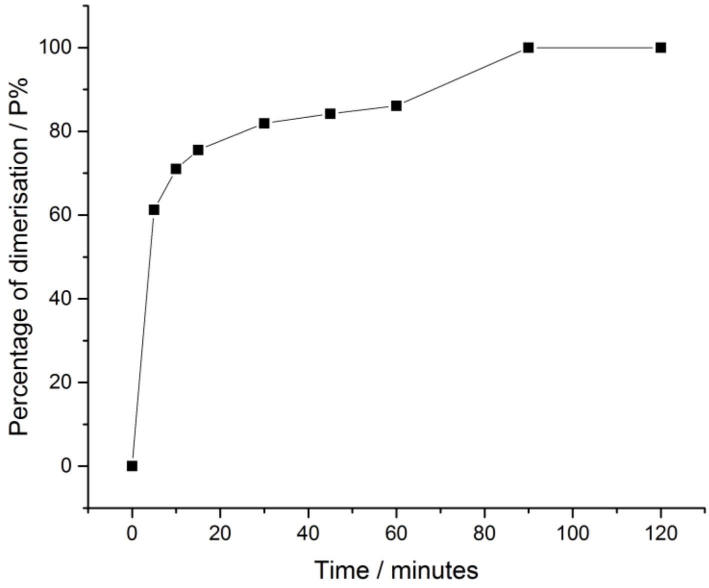

Figure S20 Time dimerization plot of **3.** Since the rate is very fast (it reached 71% in ~5 minutes), it was difficult to get the points for ln A graph to determine the rate.

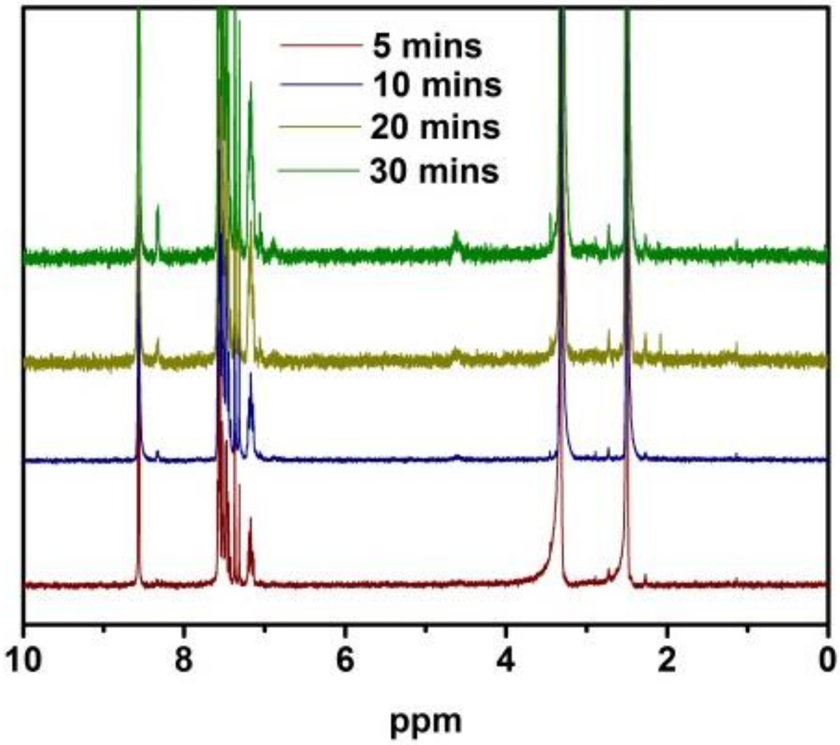

Figure S21 Time dimerization plot of **3** at 50% intensity of UV. UV irradiation was done using Asahi MAX-150 at 50% of the light intensity.

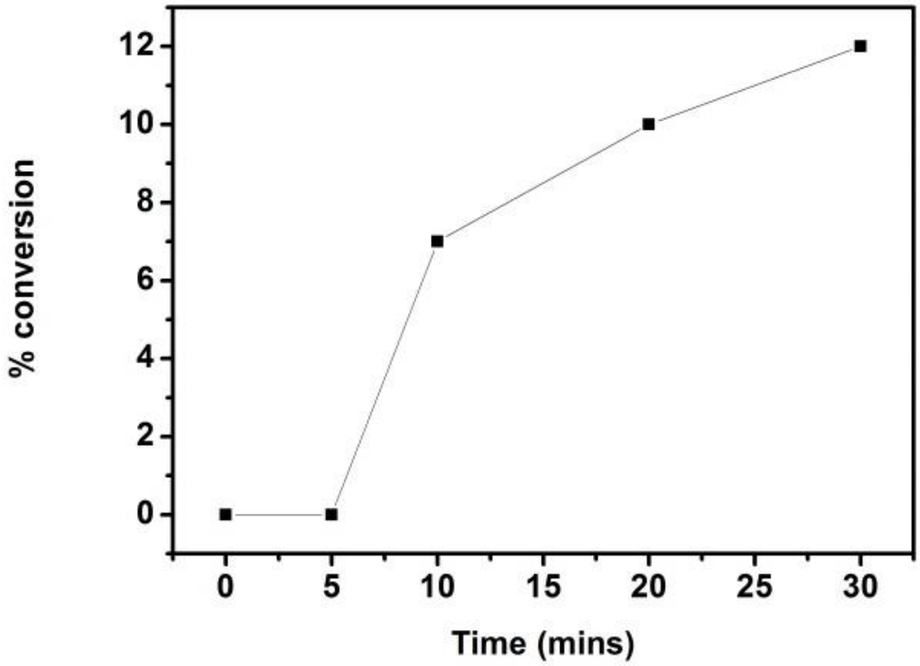

Figure S22 Percentage conversion of **3** at 50% intensity of UV.

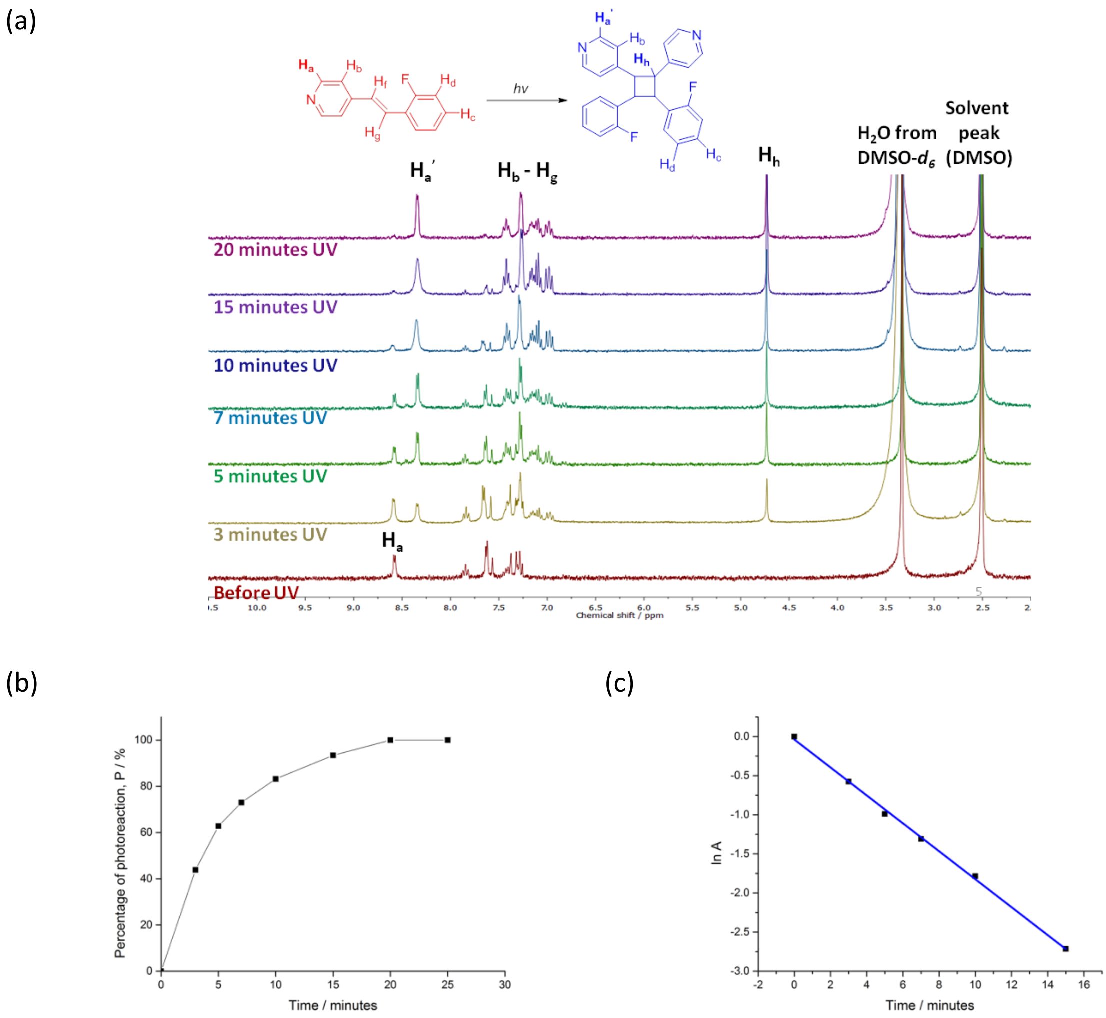

Figure S23 (a) Photocycloaddition reaction monitored through ^{1}H-NMR for 20 minutes of **4**. (b) Time-dimerisation plot of percentage dimerised product over time. (c) Graph of ln A vs time. The rate constant (k) is 0.1756 min^{-1}.

Video S1. Popping of single crystals of **4** under UV light. Recorded using normal cell phone camera.

Video S2. Popping of powdered sample of **4** under UV light. Recorded using normal cell phone camera.

References

1. Sheldrick, G. M., University of Göttingen, Germany, 1996.
2. Sheldrick, G. M. A short history of SHELX. *Acta Crystallogr. Sect. A.* **64**, 112-122 (2008).
3. Müller, P., Herbst-Irmer, R., L.Spek, A., R.Schneider, T. & R.Sawaya, M. *Crystal Structure Refinement: A Crystallographer's Guide to SHELXL*. Oxford University Press., 2006.
4. A. Spek, *J. Appl. Crystallogr.* **2003**, *36*, 7.

Crystal Data for the Bent Crystals and more Photographs:

The intensity data was measured for a bent crystal, structure was solved and refined.

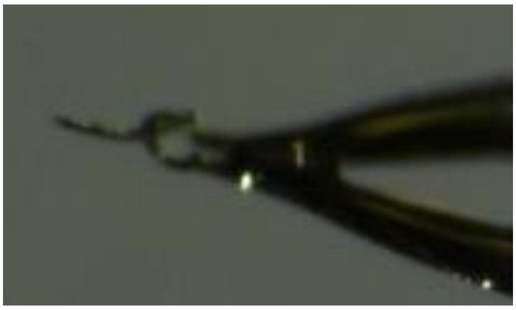

Figure S24 Bent crystal used for data collection.

Table S2 Crystal data and structure refinement for f144bi.

Empirical formula, weight	$C_{28}H_{20}F_2N_4S_2Zn$, 579.97	
Temperature	100(2) K	
Wavelength	1.54178 Å	
Crystal system, space group	Monoclinic, P 2/n	
Unit cell dimensions	a = 14.890(9) Å	α= 90°
	b = 4.935(3) Å	β= 93.507(12)°
	c = 20.286(11) Å	γ = 90°
Volume & Z	1487.9(15) Å$^{3-, 2}$	
Density (calculated)	1.295 Mg/m^3	
Absorption coefficient	2.749 mm^{-1}	
F(000)	592	
Theta range for data collection	15.059 to 56.801°	
Index ranges	-16<=h<=14, -3<=k<=5, -17<=l<=22	
Reflections collected	2988	
Independent reflections	1872 [R(int) = 0.2220]	
Completeness to theta = 67.679°	69.9 %	
Refinement method	Full-matrix least-squares on F^2	
Data / restraints / parameters	1872 / 0 / 169	
Goodness-of-fit on F^2	1.062	
Final R indices [I > 2sigma(I)]	R1 = 0.1237, wR2 = 0.3379	
R indices (all data)	R1 = 0.2680, wR2 = 0.4112	
Extinction coefficient	0.007(4)	
Largest diff. peak and hole	0.826 and -0.650 e.Å^{-3}	

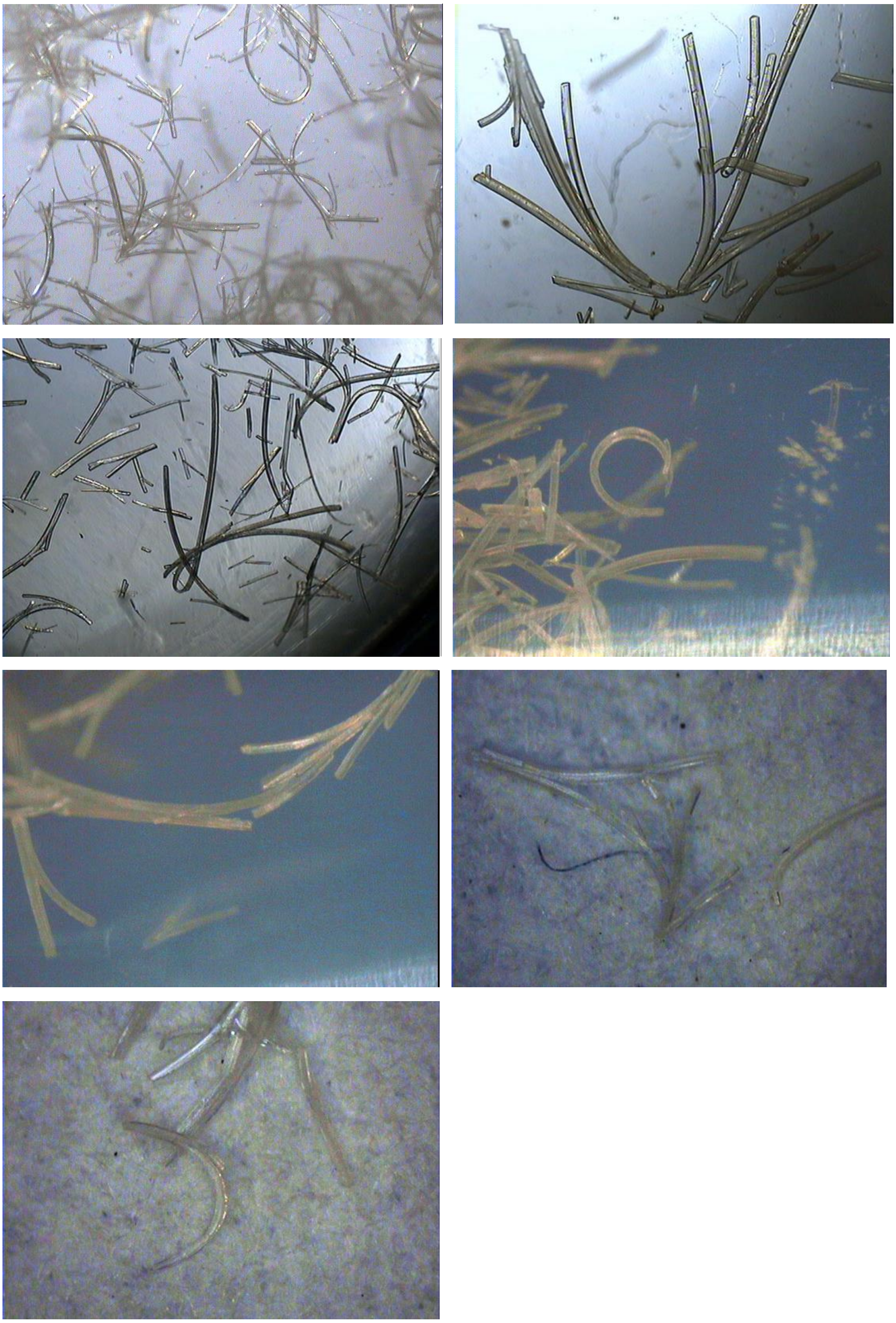

Figure S25 A few photographs of the bent crystals obtained from different crystallization conditions.
